# Risk factors for hemorrhagic transformation after tenecteplase therapy in acute ischemic stroke: A retrospective observational study

**DOI:** 10.1097/MD.0000000000050666

**Published:** 2026-09-25

**Authors:** Xiaoxuan Wang, Shaoyue Huang, Dongxu Li, Xiaowei Qiu, Zhen Hong

**Affiliations:** a The Second Department of Cerebrovascular Disease, Cangzhou Central Hospital, Cangzhou, Hebei Province, China; b Department of Neurophysiology, Cangzhou Central Hospital, Cangzhou, Hebei Province, China; c Department of Neurovascular Intervention, Cangzhou Central Hospital, Cangzhou, Hebei Province, China.

**Keywords:** acute ischemic stroke, D-dimer, hemorrhagic transformation, National Institutes of Health Stroke Scale, tenecteplase–tissue plasminogen activator

## Abstract

This retrospective observational study evaluated the incidence, subtypes, and risk factors for hemorrhagic transformation (HT) after tenecteplase–tissue plasminogen activator (TNK-tPA) therapy in patients with acute ischemic stroke. Adults with acute ischemic stroke who received intravenous TNK-tPA between June 2022 and June 2025 and had complete baseline data and follow-up neuroimaging were included. All patients underwent routine follow-up computed tomography or magnetic resonance imaging at 24 ± 6 hours after thrombolysis, with additional earlier imaging when neurological deterioration occurred. Demographic characteristics, vascular risk factors, baseline neurological severity assessed by the National Institutes of Health Stroke Scale, blood pressure, pre-stroke antiplatelet use, onset-to-needle time, Alberta Stroke Program Early CT Score, and admission laboratory parameters – including white blood cell count, hemoglobin, platelet count, international normalized ratio, fibrinogen, and D-dimer (mg/L fibrinogen-equivalent units) – were collected. HT was classified as hemorrhagic infarction (HI-1, HI-2) or parenchymal hematoma (PH-1, PH-2) according to European Cooperative Acute Stroke Study criteria. Among 181 TNK-tPA-treated patients, 28 (15.5%) developed HT, including 17 cases of HI and 11 of PH. In an exploratory multivariable logistic regression model, older age, higher baseline National Institutes of Health Stroke Scale, lower Alberta Stroke Program Early CT Score, higher systolic blood pressure, and increased international normalized ratio and D-dimer levels were associated with HT. The model showed acceptable apparent calibration and discrimination (area under the receiver operating characteristic curve = 0.86); however, 14 candidate variables were evaluated for only 28 events (approximately 2 events per variable), creating a substantial risk of overfitting. These findings are hypothesis-generating and require validation in larger independent cohorts.

## 1. Introduction

Intravenous thrombolysis remains a cornerstone of reperfusion therapy for patients with acute ischemic stroke (AIS), particularly when endovascular thrombectomy is not available or feasible. For several decades, alteplase has been the standard thrombolytic agent recommended by major guidelines. However, tenecteplase (TNK-tPA), a genetically modified tissue plasminogen activator with greater fibrin specificity and a longer half-life, has emerged as a practical alternative because it can be administered as a single intravenous bolus and may offer logistical and economic advantages in time-sensitive stroke care. Recent randomized trials and meta-analyses have shown that tenecteplase at a dose of 0.25 mg/kg is at least non-inferior, and in some analyses potentially superior, to alteplase with respect to early recanalization and functional outcomes, without a clear excess of symptomatic intracranial hemorrhage (sICH).^[1,2]^ These data have led contemporary European and international guidelines to endorse tenecteplase 0.25 mg/kg as an acceptable or preferred alternative to standard-dose alteplase for eligible AIS patients.^[3]^ Hemorrhagic transformation (HT) remains one of the most important safety concerns after intravenous thrombolysis. HT encompasses a spectrum of radiological entities, ranging from petechial hemorrhagic infarction (HI) to space-occupying parenchymal hematoma (PH), conventionally categorized according to the European Cooperative Acute Stroke Study (ECASS) criteria.^[4]^ While minor HI may be clinically silent, PH – particularly the PH-2 subtype – is strongly associated with early neurological deterioration and poor functional outcome. Reperfusion of severely ischemic tissue can trigger blood–brain barrier disruption, endothelial injury, intravascular coagulation, and extravasation of blood products, a process that can be amplified by systemic hemodynamic stress and alterations in coagulation and fibrinolysis. Recent experimental and clinical work has highlighted the roles of endothelial dysfunction, inflammatory activation, and microvascular damage in the pathogenesis of HT following recanalization therapies.^[4]^

A number of clinical and imaging predictors of HT after reperfusion therapy have been identified in alteplase-based or mixed cohorts. Meta-analytic data indicate that advanced age, higher baseline National Institutes of Health Stroke Scale (NIHSS) scores, and larger infarct burden quantified by lower Alberta Stroke Program Early CT Score (ASPECTS) or increased core volume are consistently associated with a higher risk of HT.^[5]^ Elevated baseline blood pressure, atrial fibrillation, and hyperglycemia have also been implicated as important contributors, likely reflecting more extensive ischemic injury, impaired autoregulation, and greater vascular vulnerability.^[6,7]^ In parallel, growing attention has been directed to hemostatic and fibrinolytic biomarkers. Studies of recombinant tissue plasminogen activator (rt-PA) cohorts have reported associations between low fibrinogen or platelet counts, prolonged coagulation times, and an increased risk of intracerebral hemorrhage, while post-thrombolytic or admission D-dimer elevation has been linked to sICH and unfavorable functional outcomes.^[8]^ Despite the rapid uptake of tenecteplase in many stroke systems of care, data specifically characterizing HT risk and its determinants in TNK-treated populations remain relatively limited. Most existing risk models were derived in alteplase-era cohorts and may not fully capture the hemostatic profile associated with tenecteplase, which differs from alteplase in its interaction with the coagulation and fibrinolytic systems.^[9]^ In addition, few studies have integrated clinical severity indices, standardized imaging markers such as ASPECTS, and coagulation or fibrinolytic parameters into a single multivariable framework focused on HT after TNK-tPA. Given the clinical impact of PH and the ongoing transition toward tenecteplase-based thrombolysis, there is a need for more detailed characterization of HT incidence, subtype distribution, and associated risk factors in routine practice.

Against this background, the present study investigates HT in a real-world cohort of AIS patients treated with TNK-tPA. By systematically assessing clinical variables, baseline neurological severity, early ischemic changes on computed tomography (CT), cardiovascular comorbidities such as atrial fibrillation, and selected hematologic and coagulation markers, this work aims to delineate a multifactorial risk profile for HT and to construct a predictive model with acceptable calibration and discrimination. Such information may help refine risk stratification, support individualized decision-making, and inform monitoring strategies in patients receiving tenecteplase for AIS.

Against this background, the present study investigates HT in a real-world cohort of patients with AIS treated with TNK-tPA. By systematically assessing clinical variables, baseline neurological severity, early ischemic changes on CT, cardiovascular comorbidities such as atrial fibrillation, and selected hematologic and coagulation markers, this work aims to characterize a multifactorial risk profile for HT and to explore an in-sample multivariable risk model. Given the limited number of events, model performance is reported descriptively and requires validation before clinical application. Such information may help generate hypotheses for future risk-stratification studies and inform post-thrombolysis monitoring research.

## 2. Materials and methods

### 2.1. Study design

This retrospective observational study included patients diagnosed with AIS who received TNK-tPA therapy at our institution between June 2022 and June 2025. Eligible patients were identified through electronic medical records. Inclusion criteria were age ≥18 years; diagnosis of AIS confirmed by clinical presentation and neuroimaging; administration of TNK-tPA within the recommended therapeutic time window; and availability of complete baseline clinical data and follow-up imaging for assessment of HT. Exclusion criteria were intracranial hemorrhage or hemorrhagic stroke identified on initial neuroimaging; receipt of endovascular thrombectomy before the initial post-thrombolysis imaging assessment; preexisting coagulopathy or use of anticoagulants with uncorrected abnormal coagulation parameters; severe systemic infection or another acute medical condition that could independently influence hemorrhagic risk; and incomplete clinical records or missing outcome data. The study protocol was approved by the Ethics Committee of Cangzhou Central Hospital (approval no. 2025 NO. 0178). The requirement for written informed consent was waived by the Ethics Committee because this was a retrospective analysis of de-identified, routinely collected clinical data and involved no additional intervention. The study was conducted in accordance with the Declaration of Helsinki.

### 2.2. Treatment protocol

All patients received intravenous tenecteplase in accordance with national and international guideline-recommended protocols for thrombolytic therapy in AIS. Tenecteplase was administered as a single intravenous bolus at 0.25 mg/kg, with a maximum dose of 25 mg. Before treatment, all patients underwent rapid neurological evaluation, baseline non-contrast head CT or magnetic resonance imaging (MRI) to exclude intracranial hemorrhage, and routine laboratory testing, including complete blood count, coagulation profile, and serum chemistries. Blood pressure was controlled to ≤185/110 mm Hg before drug administration and continuously monitored during the first 24 hours. After tenecteplase administration, patients were admitted to a dedicated stroke unit or intensive care unit for standardized post-thrombolysis monitoring. Neurological status was assessed at regular intervals using the NIHSS, and blood pressure, oxygen saturation, and electrocardiography were continuously monitored. All patients underwent routine follow-up neuroimaging (CT or MRI) at 24 ± 6 hours after thrombolysis. Additional imaging was performed immediately if neurological deterioration occurred; such earlier imaging did not replace the routine 24-hour examination. Antiplatelet or anticoagulant therapy was withheld during the first 24 hours and initiated only after follow-up imaging confirmed the absence of intracranial hemorrhage. Supportive management, including glycemic control, temperature regulation, and hydration, was provided according to established stroke care guidelines.

### 2.3. Data collection

Clinical, imaging, and laboratory data were extracted retrospectively from the institutional electronic medical record system using a standardized data collection framework. Demographic variables included age and sex. Clinical information comprised vascular risk factors (hypertension, diabetes mellitus, dyslipidemia, atrial fibrillation, prior stroke or transient ischemic attack), baseline neurological severity assessed by the NIHSS, pre-stroke antiplatelet use, admission systolic and diastolic blood pressure, and onset-to-needle time. Neuroimaging data were obtained from baseline non-contrast head CT performed before TNK-tPA administration. Early ischemic changes were quantified using the ASPECTS. HT was assessed on the routine follow-up CT or MRI obtained at 24 ± 6 hours after thrombolysis, together with any additional clinically indicated earlier scans, and was categorized as HI-1/HI-2 or PH-1/PH-2 according to the ECASS definitions. The incidence and distribution of HT subtypes were recorded for all patients. Laboratory indicators collected at admission included white blood cell (WBC) count, neutrophil count, lymphocyte count, hemoglobin concentration, platelet count, international normalized ratio (INR), fibrinogen level, and D-dimer concentration, reported in mg/L fibrinogen-equivalent units. All laboratory tests were processed using standardized hospital assays, and the values measured closest to the time of thrombolysis were used for analysis. ASPECTS was assessed from baseline CT imaging; however, independent double reading and formal interobserver agreement analysis were not performed.

### 2.4. Statistical analysis

All statistical procedures were conducted using IBM SPSS Statistics, version 26.0 (IBM Corp.). Continuous variables were assessed for normality and are presented as mean ± standard deviation. Comparisons between patients with and without HT were conducted using the Welch *t* test for continuous variables and the chi-square test for categorical variables. Variables with a *P* value < .10 in univariate analysis were entered into a multivariable logistic regression model. Multicollinearity among candidate predictors was assessed using variance inflation factors (VIFs); a VIF ≥5 was prespecified as indicating potentially important collinearity. For each variable in the model, regression coefficients, standard errors, Wald statistics, odds ratios (ORs), and corresponding 95% confidence intervals were reported. Model calibration was assessed using the Hosmer–Lemeshow goodness-of-fit test, and apparent in-sample discrimination was evaluated using the area under the receiver operating characteristic curve (AUC). Nagelkerke *R*^2^ was used to estimate the proportion of variance explained by the model. Fourteen candidate variables were evaluated for 28 HT events, corresponding to approximately 2 events per variable, well below the conventional 10-events-per-variable benchmark. Therefore, the multivariable analysis and model-performance estimates were considered exploratory and potentially unstable; no penalization, bootstrapping, or external validation was performed. A two-tailed *P* value < .05 was considered statistically significant.

## 3. Results

### 3.1. Baseline characteristics of the study population

A total of 181 patients with AIS treated with TNK-tPA were included in the study. The cohort consisted mainly of older adults, with a slightly higher proportion of male patients. Common vascular comorbidities in the population included hypertension, diabetes mellitus, and dyslipidemia. Atrial fibrillation and previous cerebrovascular events were also present in part of the cohort. Most patients received thrombolytic therapy within the recommended therapeutic window, and baseline neurological impairment varied across the population. Blood pressure at admission was generally elevated, which is typical in the acute phase of ischemic stroke. Neuroimaging before thrombolysis showed that the majority of patients had relatively preserved ASPECTS scores. Baseline laboratory values, including WBC count, hemoglobin, and platelet count, were within clinically acceptable ranges for thrombolytic therapy eligibility (Table 1).

**Table 1:** Baseline characteristics of the overall study population.

Characteristic	Value
Demographics	
Age, yr, mean ± SD	67.9 ± 9.2
Sex, male, n (%)	110 (60.8)
Medical history	
Hypertension, n (%)	122 (67.4)
Diabetes mellitus, n (%)	51 (28.2)
Dyslipidemia, n (%)	65 (35.9)
Atrial fibrillation, n (%)	53 (29.3)
Clinical presentation	
Baseline NIHSS score, mean ± SD	9.7 ± 4.4
Onset-to-needle time, min, mean ± SD	142.4 ± 36.9
Imaging	
ASPECTS score, mean ± SD	7.9 ± 1.2
Basic laboratory values	
WBC, ×10^9^/L, mean ± SD	8.6 ± 2.5
Hemoglobin, g/L, mean ± SD	134 ± 13
Platelets, ×10^9^/L, mean ± SD	232 ± 52

ASPECTS = Alberta Stroke Program Early CT Score, NIHSS = National Institutes of Health Stroke Scale, SD = standard deviation, WBC = white blood cell.

### 3.2. Incidence and subtypes of HT

Among the 181 patients treated with TNK-tPA, HT was identified in 28 patients, corresponding to an overall incidence of 15.5% (28/181). Based on the ECASS classification, 17 patients exhibited HI, including HI-1 in 10 cases (35.7%) and HI-2 in 7 cases (25.0%). PH was observed in 11 patients, consisting of PH-1 in 6 cases (21.4%) and PH-2 in 5 cases (17.9%). Detailed subtype distribution is presented in Table 2.

**Table 2:** Incidence and subtypes of hemorrhagic transformation.

HT category	Subtype	Definition (ECASS)	n (%)
Overall HT incidence	–	–	28/181 (15.5)
HI	HI-1	Small petechiae along the infarct margins	10 (35.7)
	HI-2	Confluent petechiae without mass effect	7 (25.0)
PH	PH-1	Hemorrhage involving <30% of infarct area with slight mass effect	6 (21.4)
	PH-2	Hemorrhage involving ≥30% of infarct area with mass effect	5 (17.9)

ECASS = European Cooperative Acute Stroke Study, HI = hemorrhagic infarction, HT = hemorrhagic transformation, PH = parenchymal hematoma.

### 3.3. Univariate analysis of risk factors for HT

Patients who developed HT were older and exhibited more severe baseline neurological deficits. The HT group had higher age (73.2 ± 8.2 vs 66.8 ± 8.7 years, *P* < .001) and NIHSS scores (14.0 ± 4.5 vs 9.0 ± 4.0, *P* < .001). Baseline systolic blood pressure (SBP) was also higher among HT patients (171.0 ± 18.3 vs 162.0 ± 16.5 mm Hg, *P* = .020). Atrial fibrillation (57.1% vs 24.2%, *P* < .001) and pre-stroke antiplatelet use (42.9% vs 22.2%, *P* = .021) were significantly associated with HT. Imaging analysis showed lower ASPECTS in the HT group (7.3 ± 1.0 vs 8.1 ± 1.2, *P* < .001). Among laboratory parameters, elevated WBC (*P* = .009), neutrophils (*P* = .004), D-dimer (*P* < .001), longer INR (*P* = .027), lower lymphocyte count (*P* = .002), and lower hemoglobin (*P* = .012) were associated with HT. Fibrinogen demonstrated a borderline association (*P* = .058), while platelet count showed no significant difference (*P* = .345). Variables with *P* < .10 (age, sex, NIHSS, SBP, ASPECTS, atrial fibrillation, antiplatelet use, WBC, neutrophils, lymphocytes, hemoglobin, INR, fibrinogen, and D-dimer) were included in the multivariate logistic regression (Table 3).

**Table 3:** Univariate analysis comparing HT versus non-HT groups.

Variable	HT (n = 28)	Non-HT (n = 153)	Test	Statistic	*P* value
Demographic/clinical variables					
Age, yr	73.2 ± 8.2	66.8 ± 8.7	Welch *t*	*t* = 3.76	<.001
Sex, male, n (%)	12 (42.9)	98 (64.1)	χ^2^	χ^2^ = 4.46	.035
Baseline NIHSS score	14.0 ± 4.5	9.0 ± 4.0	Welch *t*	*t* = 5.50	<.001
Onset-to-needle time, min	149.0 ± 35.6	141.0 ± 37.3	Welch *t*	*t* = 1.07	.285
Baseline SBP, mm Hg	171.0 ± 18.3	162.0 ± 16.5	Welch *t*	*t* = 2.43	.020
Baseline DBP, mm Hg	96.0 ± 12.0	92.0 ± 11.0	Welch *t*	*t* = 1.64	.109
Atrial fibrillation, n (%)	16 (57.1)	37 (24.2)	χ^2^	χ^2^ = 12.42	<.001
Hypertension, n (%)	21 (75.0)	101 (66.0)	χ^2^	χ^2^ = 0.87	.351
Diabetes mellitus, n (%)	10 (35.7)	41 (26.8)	χ^2^	χ^2^ = 0.74	.390
Pre-stroke antiplatelet use, n (%)	12 (42.9)	34 (22.2)	χ^2^	χ^2^ = 5.32	.021
Imaging variable					
ASPECTS score	7.3 ± 1.0	8.1 ± 1.2	Welch *t*	*t* = –3.61	<.001
Laboratory variables (8 selected indicators)					
WBC, ×10^9^/L	9.8 ± 2.7	8.4 ± 2.3	Welch *t*	*t* = 2.71	.009
Neutrophils, ×10^9^/L	7.1 ± 2.4	5.8 ± 2.0	Welch *t*	*t* = 2.98	.004
Lymphocytes, ×10^9^/L	1.3 ± 0.5	1.7 ± 0.6	Welch *t*	*t* = –3.23	.002
Hemoglobin, g/L	128 ± 14	135 ± 13	Welch *t*	*t* = –2.59	.012
Platelets, ×10^9^/L	224 ± 53	234 ± 51	Welch *t*	*t* = –0.95	.345
INR	1.10 ± 0.12	1.05 ± 0.10	Welch *t*	*t* = 2.25	.027
Fibrinogen, g/L	3.1 ± 0.8	3.4 ± 0.9	Welch *t*	*t* = –1.93	.058
D-dimer, mg/L FEU	1.8 ± 1.0	1.1 ± 0.8	Welch *t*	*t* = 3.64	<.001

ASPECTS = Alberta Stroke Program Early CT Score, DBP = diastolic blood pressure, FEU = fibrinogen-equivalent units, HT = hemorrhagic transformation, INR = international normalized ratio, NIHSS = National Institutes of Health Stroke Scale, SBP = systolic blood pressure, WBC = white blood cell count.

### 3.4. Multicollinearity diagnostics

Multicollinearity diagnostics were performed for all 14 candidate predictors entered into the multivariable model. The VIF values ranged from 1.10 to 4.21, and tolerance values ranged from 0.238 to 0.909. The highest VIF was observed for neutrophil count (VIF = 4.21), followed by WBC count (VIF = 3.46), reflecting the expected correlation between total leukocyte and neutrophil measurements. Nevertheless, no candidate predictor exceeded the prespecified VIF threshold of 5, and all tolerance values were greater than 0.20. These findings indicated that potentially important multicollinearity was not detected among the candidate variables (Table 4).

**Table 4:** Multivariable logistic regression analysis.

Variable	β	SE	Wald	*P* value	OR	95% CI for OR
Age (per 1-yr increase)	0.058	0.028	4.33	.037	1.06	1.00–1.12
Male sex (male vs female)	−0.511	0.450	1.29	.256	0.60	0.25–1.45
Baseline NIHSS (per 1-point increase)	0.131	0.060	4.77	.029	1.14	1.01–1.28
Baseline SBP (per 10-mm Hg increase)	0.247	0.120	4.23	.040	1.28	1.01–1.62
ASPECTS (per 1-point increase)	−0.342	0.160	4.58	.032	0.71	0.52–0.97
Atrial fibrillation (yes vs no)	1.065	0.550	3.75	.053	2.90	0.99–8.52
Pre-stroke antiplatelet use (yes vs no)	0.668	0.500	1.78	.182	1.95	0.73–5.20
WBC (per 1 × 10^9^/L increase)	0.113	0.080	2.01	.157	1.12	0.96–1.31
Neutrophils (per 1 × 10^9^/L increase)	0.039	0.090	0.19	.663	1.04	0.87–1.24
Lymphocytes (per 1 × 10^9^/L increase)	−0.545	0.380	2.05	.152	0.58	0.28–1.22
Hemoglobin (per 1 g/L increase)	−0.020	0.015	1.81	.178	0.98	0.95–1.01
INR (per 0.1-unit increase)	0.166	0.070	5.59	.018	1.18	1.03–1.35
Fibrinogen (per 1 g/L increase)	−0.223	0.200	1.24	.265	0.80	0.54–1.18
D-dimer (per 1 mg/L FEU increase)	0.470	0.180	6.82	.009	1.60	1.12–2.28

ASPECTS = Alberta Stroke Program Early CT Score, CI = confidence interval, FEU = fibrinogen-equivalent units, INR = international normalized ratio, NIHSS = National Institutes of Health Stroke Scale, OR = odds ratio, SBP = systolic blood pressure, SE = standard error, WBC = white blood cell, Wald = Wald χ² statistic, β = regression coefficient.

### 3.5. Multivariable logistic regression for HT after TNK-tPA

In the exploratory multivariable model, including all candidate covariates from univariate screening, age, baseline neurological severity, early ischemic burden on imaging, baseline SBP, coagulation status, and fibrinolytic activation were associated with HT. Specifically, each 1-year increase in age was associated with higher odds of HT (OR = 1.06, *P* = .037). Baseline NIHSS was associated with HT (OR = 1.14 per point, *P* = .029), while higher ASPECTS was associated with lower odds (OR = 0.71 per point, *P* = .032). Baseline SBP showed a graded association (OR = 1.28 per 10-mm Hg increase, *P* = .040). INR (per 0.1 unit) and D-dimer were also associated with HT (OR = 1.18, *P* = .018; OR = 1.60, *P* = .009, respectively). Atrial fibrillation showed a borderline association (*P* = .053). Sex, pre-stroke antiplatelet use, and inflammatory cell indices (WBC, neutrophils, and lymphocytes) were not statistically significant after adjustment. Given the low events-per-variable ratio, these adjusted estimates should be interpreted cautiously rather than as definitive independent effects (Table 5).

**Table 5:** Multicollinearity diagnostics for candidate predictors included in the multivariable model.

Predictor	Tolerance	VIF	Interpretation
Age	0.704	1.42	No important multicollinearity
Sex, male	0.909	1.10	No important multicollinearity
Baseline NIHSS score	0.595	1.68	No important multicollinearity
Baseline systolic blood pressure	0.806	1.24	No important multicollinearity
ASPECTS	0.629	1.59	No important multicollinearity
Atrial fibrillation	0.730	1.37	No important multicollinearity
Pre-stroke antiplatelet use	0.847	1.18	No important multicollinearity
WBC count	0.289	3.46	Moderate correlation, below threshold
Neutrophil count	0.238	4.21	Highest VIF, below threshold
Lymphocyte count	0.581	1.72	No important multicollinearity
Hemoglobin	0.763	1.31	No important multicollinearity
INR	0.820	1.22	No important multicollinearity
Fibrinogen	0.735	1.36	No important multicollinearity
D-dimer	0.671	1.49	No important multicollinearity

VIF ≥5 or tolerance ≤0.20 was considered suggestive of potentially important multicollinearity.

ASPECTS = Alberta Stroke Program Early CT Score, INR = international normalized ratio, NIHSS = National Institutes of Health Stroke Scale, VIF = variance inflation factor, WBC = white blood cell.

### 3.6. Model performance

The exploratory multivariable logistic regression model showed acceptable apparent calibration and discrimination in the derivation sample. The Hosmer–Lemeshow test showed no statistically significant deviation between predicted and observed HT risk (χ^2^ = 6.21, df = 8, *P* = .624), and the Nagelkerke *R*^2^ was 0.41. The apparent AUC was 0.86 (95% confidence interval = 0.79–0.93; Table 6). However, because 14 candidate variables were evaluated for only 28 events and no internal or external validation was performed, these in-sample performance estimates are likely optimistic and should not be interpreted as evidence of a validated prediction model.

**Table 6:** Multivariable logistic regression analysis.

Variable	β	SE	Wald	*P* value	OR	95% CI for OR
Age (per 1-yr increase)	0.058	0.028	4.33	.037	1.06	1.00–1.12
Male sex (male vs female)	−0.511	0.450	1.29	.256	0.60	0.25–1.45
Baseline NIHSS (per 1-point increase)	0.131	0.060	4.77	.029	1.14	1.01–1.28
Baseline SBP (per 10-mm Hg increase)	0.247	0.120	4.23	.040	1.28	1.01–1.62
ASPECTS (per 1-point increase)	−0.342	0.160	4.58	.032	0.71	0.52–0.97
Atrial fibrillation (yes vs no)	1.065	0.550	3.75	.053	2.90	0.99–8.52
Pre-stroke antiplatelet use (yes vs no)	0.668	0.500	1.78	.182	1.95	0.73–5.20
WBC (per 1 × 10^9^/L increase)	0.113	0.080	2.01	.157	1.12	0.96–1.31
Neutrophils (per 1 × 10^9^/L increase)	0.039	0.090	0.19	.663	1.04	0.87–1.24
Lymphocytes (per 1 × 10^9^/L increase)	−0.545	0.380	2.05	.152	0.58	0.28–1.22
Hemoglobin (per 1 g/L increase)	−0.020	0.015	1.81	.178	0.98	0.95–1.01
INR (per 0.1-unit increase)	0.166	0.070	5.59	.018	1.18	1.03–1.35
Fibrinogen (per 1 g/L increase)	−0.223	0.200	1.24	.265	0.80	0.54–1.18
D-dimer (per 1 mg/L FEU increase)	0.470	0.180	6.82	.009	1.60	1.12–2.28

ASPECTS = Alberta Stroke Program Early CT Score, CI = confidence interval, FEU = fibrinogen-equivalent units, INR = international normalized ratio, NIHSS = National Institutes of Health Stroke Scale, OR = odds ratio, SBP = systolic blood pressure, SE = standard error, WBC = white blood cell count, Wald = Wald χ² statistic, β = regression coefficient.

## 4. Discussion

This study evaluated the incidence, radiological subtypes, and risk factors for HT after TNK-tPA therapy in patients with AIS. The overall HT incidence of 15.5% and the predominance of HI over PH are consistent with the concept that most post-thrombolytic hemorrhagic events represent minor reperfusion-related leakage rather than large hematomas. The ECASS-based distribution, with HI-1 and HI-2 accounting for approximately two-thirds of HT events and PH-1/PH-2 comprising the remainder, reflects a spectrum of vascular injury ranging from petechial extravasation to space-occupying hematoma. Clinically, this pattern is relevant because PH, especially PH-2, is more often associated with early neurological deterioration and poor outcome, whereas small petechial HI may have limited functional impact. In the exploratory multivariable analysis, age, baseline NIHSS score, ASPECTS, SBP, INR, and D-dimer were associated with HT, while atrial fibrillation showed a borderline association. Given the low events-per-variable ratio, these findings should not be interpreted as definitive independent determinants. Collectively, the observed signals span 3 interrelated domains: patient frailty and vascular vulnerability (age), the extent and severity of ischemic injury (NIHSS and ASPECTS), and hemodynamic and hemostatic balance (blood pressure, INR, and D-dimer). Older age may contribute to structural vessel-wall changes, impaired autoregulation, and increased susceptibility of the blood–brain barrier to reperfusion injury. Higher NIHSS and lower ASPECTS are markers of larger ischemic areas with more severe metabolic failure and endothelial damage, which provide a substrate for hemorrhagic conversion following recanalization.

Elevated SBP at presentation may aggravate mechanical stress on damaged microvasculature at the time of reperfusion, favoring extravasation of blood into infarcted tissue. The observed associations of INR and D-dimer with HT may indicate that reduced coagulation reserve and enhanced fibrinolytic activation contribute to bleeding risk. Patients with higher INR have a reduced capacity to generate stable fibrin clots, whereas elevated D-dimer reflects active fibrin degradation and increased fibrinolytic activity. In this context, TNK-tPA could further shift the hemostatic balance toward bleeding in predisposed patients. Although inflammatory markers, including WBC count, neutrophil count, and lymphocyte count, were associated with HT in univariate analysis, they were not statistically significant after multivariable adjustment. This suggests that their crude associations may partly reflect stroke severity and early ischemic burden. Likewise, pre-stroke antiplatelet use was associated with HT in unadjusted comparisons but not in the exploratory adjusted model, indicating that background antiplatelet therapy may be a marker of higher vascular risk rather than a direct causal factor when thrombolysis is administered within guideline-recommended indications.

The present findings align closely with recent evidence on predictors of HT after intravenous reperfusion therapy. A 2023 systematic review and meta-analysis by Sun et al identified advanced age, higher baseline NIHSS scores, lower ASPECTS, and atrial fibrillation as consistent risk factors for HT following intravenous thrombolysis or endovascular therapy, supporting the central role of patient age, stroke severity, and ischemic core extent in bleeding risk.^[5]^ Similarly, a 2023 and 2024 body of work examining early HT after reperfusion therapy reported that patients with larger infarcts and higher NIHSS at onset had substantially higher rates of PH, and that AF frequently co-occurred with these features rather than acting as an independent driver.^[10]^ Blood pressure-related parameters have also been emphasized in recent reviews. Zubair et al summarized observational data showing that elevated SBP and greater blood pressure variability are associated with increased risk of HT and sICH after thrombolysis, emphasizing careful hemodynamic management in the hyperacute phase.^[11]^ The graded relationship between SBP and HT observed in the current study is concordant with these findings and reinforces the position that blood pressure is a modifiable risk factor in the peri-thrombolytic period.

Coagulation and fibrinolytic markers have become a focus of recent predictive research. Several studies have reported that elevated baseline or follow-up D-dimer levels are associated with sICH and poor functional outcome after intravenous rt-PA or mechanical thrombectomy. Qiu et al demonstrated that high admission D-dimer predicted symptomatic hemorrhage after thrombectomy, while Qiu et al showed that post-thrombolytic D-dimer elevation was linked to both symptomatic hemorrhage and unfavorable 3-month outcomes.^[12]^ More recently, Li et al proposed composite indices such as the D-dimer/albumin ratio as novel predictors of hemorrhagic complications and prognosis in acute cerebrovascular disease, further supporting the prognostic value of fibrin turnover markers.^[13]^ The independent association between D-dimer and HT in this TNK-tPA cohort is therefore consistent with, and extends, these observations into the specific context of tenecteplase-based thrombolysis. The observed association between D-dimer and HT in this TNK-tPA cohort is therefore consistent with these reports, although confirmation in a larger validated model is required before concluding that it is an independent TNK-tPA-specific predictor.

The present findings are also compatible with contemporary work on HT prediction models. Recent logistic regression- and machine-learning-based models frequently incorporate age, NIHSS, ASPECTS, blood pressure, and coagulation parameters as core predictors, achieving AUC values in the range of 0.80 to 0.90.^[14]^ The apparent AUC of 0.86 and Nagelkerke *R*^2^ of 0.41 fall within this performance range; however, these in-sample estimates may be optimistic because of the low events-per-variable ratio and the absence of internal or external validation. Moreover, recent comparative studies of alteplase and tenecteplase have reported similar HT rates between the 2 agents, indicating that the risk profile is likely driven more strongly by patient- and lesion-level factors than by the choice of thrombolytic drug alone.^[15]^ Regarding atrial fibrillation, recent analyses have suggested that AF is associated with HT primarily through its impact on stroke phenotype – larger cardioembolic infarcts with poorer collaterals – rather than acting as an independent determinant when adjusting for these features.^[5]^ The borderline significance of AF in the present multivariable model is compatible with this interpretation and suggests that AF may still identify a high-risk subgroup, but that its effect is mediated through stroke severity, infarct size, and coexisting hemostatic disturbances.

Age, NIHSS, ASPECTS, and SBP are well-established predictors of HT across thrombolytic agents and likely represent generalizable risk factors. The observed associations of INR and D-dimer may reflect the role of coagulation and fibrinolytic balance in the context of TNK-tPA; however, they should be interpreted cautiously because of the model’s high risk of overfitting and the absence of a direct comparison with alteplase-treated cohorts.

From a clinical perspective, these findings support continued attention to integrated risk assessment before and after TNK-tPA administration. Older age, high baseline NIHSS, extensive early ischemia (low ASPECTS), elevated SBP, and abnormal hemostatic markers (higher INR and D-dimer) may help flag patients who warrant intensified monitoring, careful blood pressure management, and timely follow-up imaging. These factors should not be used as a standalone decision rule or as contraindications to thrombolysis. Because the model is exploratory and at substantial risk of overfitting, its potential clinical utility must be evaluated in larger, independently validated cohorts before implementation.

The present study has several strengths. First, it focuses specifically on patients treated with TNK-tPA, a thrombolytic agent that is increasingly adopted as an alternative to alteplase in AIS, thereby adding data in a still-evolving therapeutic context. Second, the analysis included a comprehensive set of clinical, imaging, and laboratory variables, allowing simultaneous evaluation of demographic, hemodynamic, ischemic burden, and hemostatic contributors to HT. Third, the use of multivariable logistic regression with assessment of model calibration and discrimination provides a methodologically sound framework for risk estimation and supports the internal validity of the findings. However, important limitations must be acknowledged. The retrospective single-center design introduces potential selection and information biases and may limit the generalizability of results to other populations or practice settings. The modest number of HT events (n = 28) constrains the number of predictors that can be reliably included in multivariable models and raises the possibility of overfitting despite cautious variable selection. The study relied on CT-based ASPECTS without advanced perfusion imaging or volumetric quantification of infarct core, which may have led to imperfect characterization of tissue at risk. Laboratory measurements were obtained at a single time point on admission; dynamic changes in coagulation or inflammatory markers were not assessed and could provide additional prognostic information. Furthermore, the study did not differentiate clinical impact according to HT subtype (e.g., HI vs PH) in outcome models, and symptomatic versus asymptomatic HT was not analyzed separately, which may be relevant for refining clinical decision-making. Finally, residual confounding by unmeasured factors, including genetic hemostasis variants and detailed antithrombotic exposure patterns, cannot be excluded. sICH was not specifically assessed in this study, as clinical deterioration criteria were not systematically incorporated into the outcome definition. Consequently, the findings are based on radiological HT rather than clinically significant hemorrhagic events, which may limit the direct applicability of the results to clinical decision-making and risk stratification.

## 5. Conclusion

In this cohort of TNK-tPA-treated AIS patients, HT occurred in 15.5%, with both HI and PH represented. Older age, greater neurological severity, lower ASPECTS, elevated SBP, and abnormalities in coagulation and fibrinolysis independently increased HT risk. These findings highlight a multifactorial risk profile that may support refined monitoring and individualized peri-thrombolysis management.

## Author contributions

**Conceptualization:** Xiaoxuan Wang, Shaoyue Huang, Dongxu Li, Xiaowei Qiu, Zhen Hong.

**Data curation:** Xiaoxuan Wang, Shaoyue Huang, Dongxu Li, Xiaowei Qiu, Zhen Hong.

**Formal analysis:** Xiaoxuan Wang, Shaoyue Huang, Dongxu Li, Zhen Hong.

**Funding acquisition:** Xiaoxuan Wang, Zhen Hong.

**Investigation:** Zhen Hong.

**Writing** – **original draft:** Xiaoxuan Wang, Xiaowei Qiu, Zhen Hong.

**Writing** – **review & editing:** Xiaoxuan Wang, Xiaowei Qiu, Zhen Hong.
